# Calcium signalling links MYC to NUAK1

**DOI:** 10.1038/onc.2017.394

**Published:** 2017-11-06

**Authors:** T Monteverde, J Tait-Mulder, A Hedley, J R Knight, O J Sansom, D J Murphy

**Affiliations:** 1 Institute of Cancer Sciences, University of Glasgow, Glasgow, Scotland, UK; 2 CRUK Beatson Institute, Garscube Estate, Glasgow, UK

## Abstract

NUAK1 is a member of the AMPK-related family of kinases. Recent evidence suggests that NUAK1 is an important regulator of cell adhesion and migration, cellular and organismal metabolism, and regulation of TAU stability. As such, NUAK1 may play key roles in multiple diseases ranging from neurodegeneration to diabetes and metastatic cancer. Previous work revealed a crucial role for NUAK1 in supporting viability of tumour cells specifically when MYC is overexpressed. This role is surprising, given that NUAK1 is activated by the tumour suppressor LKB1. Here we show that, in tumour cells lacking LKB1, NUAK1 activity is maintained by an alternative pathway involving calcium-dependent activation of PKCα. Calcium/PKCα-dependent activation of NUAK1 supports engagement of the AMPK-TORC1 metabolic checkpoint, thereby protecting tumour cells from MYC-driven cell death, and indeed, MYC selects for this pathway in part via transcriptional regulation of PKCα and ITPR. Our data point to a novel role for calcium in supporting tumour cell viability and clarify the synthetic lethal interaction between NUAK1 and MYC.

## Introduction

NUAK1, also known as ARK5, is one of 12 kinases related by sequence homology to the catalytic α-subunits of the metabolic regulator AMPK.^1^ Perturbation of NUAK1 has revealed a diverse array of phenotypes, pointing to roles for NUAK1 in regulating cell adhesion,^2^ directional migration,^3, 4^ neuronal axon branching,^5^ glycogen synthesis,^6^ replicative senescence^7^ and TAU stabilization.^8^ Overexpression of NUAK1 is associated with poor prognosis in multiple cancers, including colorectal,^9^ (Port, *et al.*, personal communication) ovarian,^10, 11, 12^ and lung,^13^ among others.^14^ Accordingly, NUAK1 is a common target of multiple miRNAs that are frequently lost during progression to metastatic disease.^15, 16, 17, 18, 19, 20^ Despite the evidence that NUAK1 may contribute to multiple diseases, the signal transduction context of NUAK1 remains poorly defined.

We previously identified a role for NUAK1 in supporting viability of cancer cells when MYC is overexpressed.^21^ Briefly, we showed that MYC-overexpressing cells are unable to maintain energetic homoeostasis in the absence of NUAK1, in part due to a failure to efficiently activate AMPK and slow TORC1-dependent protein translation when faced with metabolic stress. NUAK1 and AMPK thus protect cancer cells from metabolic stress, which is a hallmark of most solid tumours.^22, 23^ This tumour-promoting activity of NUAK1 and AMPK is somewhat paradoxical, given that both are activated by LKB1, an established tumour suppressor: LKB1 phosphorylates AMPKα subunits on Thr^172^, and NUAK1 on Thr^211^, within the conserved T-loop of the kinase domain.^1, 24^ Notably, AMPKα^T172^ is phosphorylated by CamKK2 in response to calcium signalling,^25, 26^ suggesting that the T-loops of these kinases may be accessible to other upstream regulators in addition to LKB1.

Here we demonstrate that NUAK1, like AMPK, is active in cancer cells in the absence of LKB1. Similar to AMPK, basal NUAK1 activity is maintained by tonic Ca^2+^ signalling and activity increases in response to Ca^2+^ mobilization. Unlike AMPK, NUAK1 does not appear to be regulated by CamKK2, but rather by Ca^2+^-dependent activation of PKCα. Significantly, suppression of either NUAK1 or PKCα leads to MYC-dependent cell death and MYC selects for increased Ca^2+^ signalling in part via transcriptional regulation of Ca^2+^-dependent protein kinases. Our work thus reveals a novel role for Ca^2+^ signalling in supporting viability of MYC-overexpressing cells via activation of PKCα and NUAK1.

## Results

### NUAK1 is specifically required for Ca^2+^-dependent AMPK activity

Depletion of NUAK1 impairs activation of AMPK in response to sustained MYC deregulation.^21^ We asked whether this requirement for NUAK1 is a general feature of AMPK regulation or rather a context-dependent event. AMPK is activated by phosphorylation of the α-subunit on Thr^172^ by LKB1, and activity is further enhanced upon a drop in the ATP:AMP/ADP ratio.^27^ Alternatively, CamKK2 can phosphorylate AMPKα Thr^172^ in response to calcium signalling.^25^ Additionally, AMPK can be activated upon direct binding of pharmacological agonists, such as salicylate or A769662.^28^ We therefore considered three modes of AMPK activation: indirect activation in response to energetic stress, direct activation by agonist binding and calcium-dependent activation.

**Figure 1:**
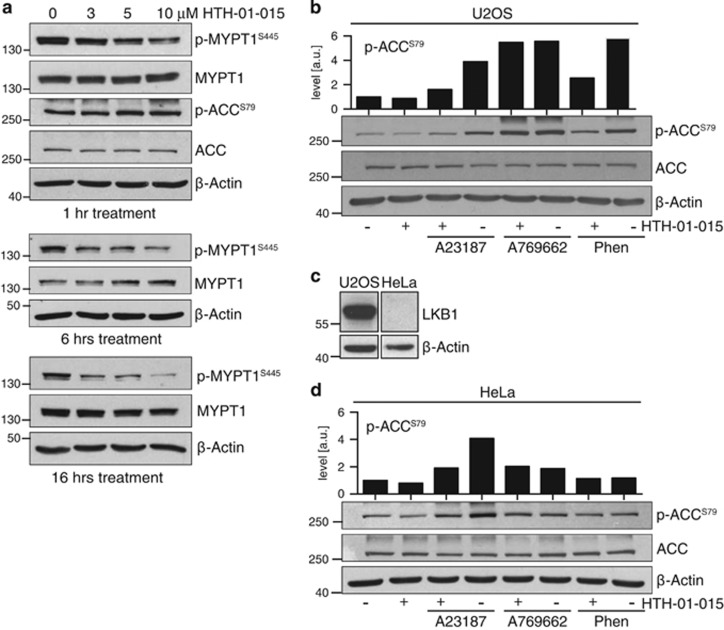
NUAK1 is required for calcium-dependent activation of AMPK. (**a**) Whole-cell extracts from U2OS cells treated with the indicated concentrations of HTH-01-015 for 1, 6 or 16 h and probed with the indicated antibodies. (**b**) Lysates from U2OS cells pre-treated with HTH-01-015 for 1 h prior to stimulation with 3 μm A23187 (10 min), 100 μm A769662 (1 h) or 10 mm Phenformin (1 h) and blotted for phospho- and total ACC. Densitometry shows p-ACC levels in the image shown. (**c**) Lysates from equal numbers of U2OS and HeLa cells were probed for LKB1. Images are from the same gel and immunoblot, but rearranged to omit extraneous data. (**d**) Lysates from HeLa cells pre-treated with HTH-01-015 for 1 h prior to stimulation with 3 μm A23187 (10 min), 100 μm A769662 (1 h) or 10 mm Phenformin (1 h) and blotted for phospho- and total ACC. Densitometry shows p-ACC levels in the image shown. All images are representative of at least three independent experiments.

In order to investigate the requirement of each mode of AMPK activation for NUAK1, we made use of a recently described highly selective NUAK1 kinase inhibitor, HTH-01-015. This molecule shows little-to-no activity towards AMPK, NUAK2 or other AMPK-related kinases (ARKs) *in vitro*.^29^ Treatment of U2OS cells with HTH-01-015 for 1 h, 6 h or overnight reduced Ser^445^ phosphorylation of the NUAK1 substrate MYPT1 in a dose-dependent manner (Figure 1a). In contrast, acute treatment with HTH-01-015 had no effect on basal Ser^79^ phosphorylation of the canonical AMPK substrate, ACC. Acute activation of AMPK in U2OS cells using the direct agonist A769662, the electron transport chain inhibitor phenformin or the widely-used Ca^2+^ ionophore A23187, all increased phospho-ACC levels. Co-treatment with HTH-01-015 attenuated this increase in response to AMPK activation by Ca^2+^ and by phenformin, but not by the direct AMPK agonist A769662, indicating that the requirement for NUAK1 during AMPK activation is context dependent (Figure 1b).

Suppression of Ca^2+^-dependent AMPK activity by HTH-01-015 suggested that NUAK1 may be important during LKB1-independent regulation of AMPK. We therefore repeated the above analysis in HeLa cells, which lack functional LKB1 (Figure 1c). As measured by ACC phosphorylation, activation of AMPK by either direct agonist or phenformin was much weaker in HeLa cells than in U2OS cells, and neither was affected by NUAK1 inhibition. In contrast, Ca^2+^ ionophore clearly increased AMPK activity and this increase was attenuated by NUAK1 inhibition (Figure 1d), suggesting a specific role for NUAK1 in this mode of AMPK activation.

### NUAK1 is activated by calcium signalling

**Figure 2:**
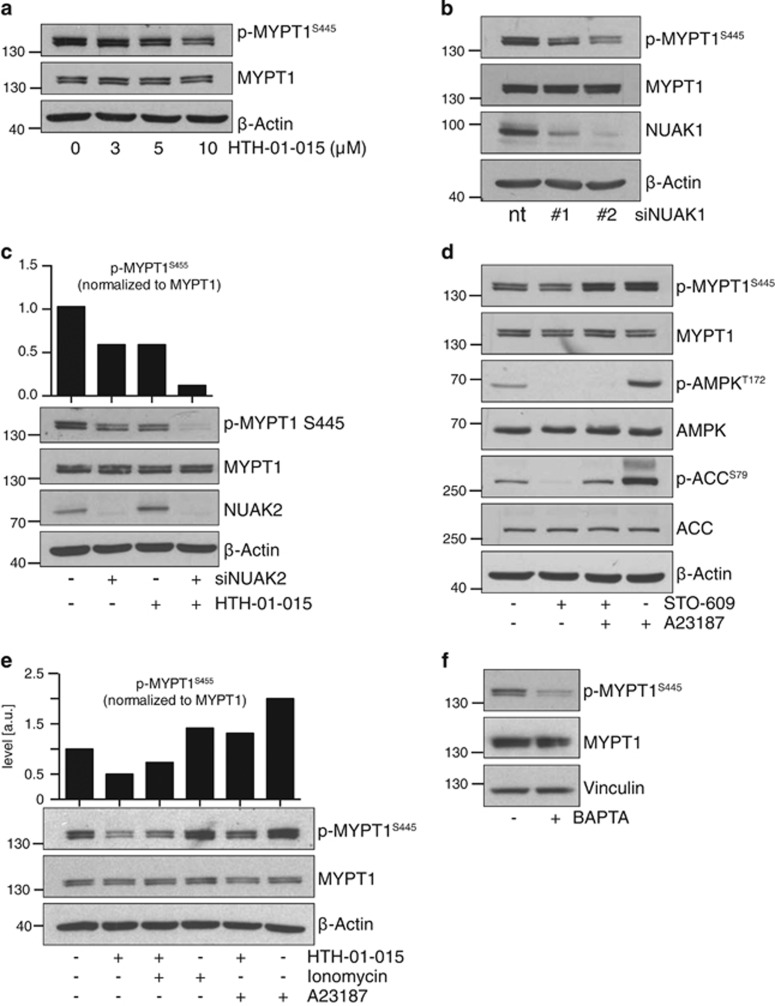
Calcium signalling activates NUAK1. (**a**) Lysates from HeLa cells treated with the indicated concentrations of HTH-01-015 for 1 h and probed for phospho- and total MYPT1. (**b**) Lysates from HeLa cells transfected with NUAK1 siRNA and probed with the indicated antibodies. nt, non-targeting control siRNA. (**c**) Lysates from HeLa cells transfected with NUAK2 (+) or control (−) siRNA and treated ±10 μm HTH-01-015, as indicated. Densitometry shows phospho-MYPT1 levels from the image shown. (**d**) Lysates from HeLa cells pre-treated with 5 μg/ml STO-609 for 1 h prior to stimulation with 3 μm A23187 (10 min) as indicated, and probed with the indicated antibodies. (**e**) Lysates from HeLa cells pre-treated with 10 μm HTH-01-015 for 1 h prior to stimulation with 3 μm A23187 or Ionomycin (both 10 min) as indicated, and probed for phosphor-MYPT1. (**f**) Lysates from HeLa cells treated ±20 μm BAPTA for 30 min. All images are representative of at least three independent experiments, except (**f**) where *N*=2.

LKB1 is a master regulator of the AMPK-related kinases, including NUAK1.^1^ Our results in HeLa cells implied that NUAK1 is active in these cells despite the absence of LKB1. The myosin phosphatase targeting subunit of PP1β, MYPT1 (PPP1R12A), is to date the best-characterized substrate of NUAK1 kinase activity.^2^ Acute treatment of HeLa cells with HTH-01-015 reduced MYPT1 phospho-Ser^445^ levels, suggesting that NUAK1 is indeed catalytically active in these cells (Figure 2a). Depletion of NUAK1 using two independent siRNAs also reduced phospho-MYPT1^S445^, confirming the specificity of the inhibitor effect (Figure 2b). The partial reduction in phospho-MYPT1^S445^ observed upon NUAK1 suppression suggested that other kinases may contribute to MYPT1^S445^ phosphorylation. Indeed, NUAK2, the ARK most closely related to NUAK1, was previously reported to phosphorylate this site.^2^ Accordingly, depletion of NUAK2 also reduced phospho-MYPT1^S445^ levels, while combined suppression of both NUAK1 and 2 almost completely abolished MYPT1^S445^ phosphorylation (Figure 2c). Pharmacological inhibition of both NUAK1 and NUAK2, using the dual-specificity inhibitor WZ4003^29^ similarly abolished MYPT1 phosphorylation, corroborating the results of the siRNA (Supplementary Figure S1A). Thus, both NUAK1 and NUAK2 are active in HeLa cells despite their LKB1-null status.

These data indicate that NUAK1 is activated in HeLa cells by an alternative upstream kinase. We first asked if CamKK2, a known upstream activator of AMPK, might similarly activate NUAK1. Treatment of HeLa cells with the CamKK2 inhibitor STO-609 strongly suppressed phosphorylation of both AMPKα^T172^ and ACC^S79^ but had no influence on phospho-MYPT1^S445^ levels (Figure 2d), suggesting that CamKK2 is not upstream of NUAK1. Strikingly, treatment with calcium ionophore A23187 increased phosphorylation of both ACC^S79^ and MYPT1^S445^ and co-treatment with STO-609 reduced ACC^S79^ phosphorylation but again had no effect on phospho-MYPT1^S445^, suggesting that NUAK1 is activated by a calcium-dependent kinase other than CamKK2. Accordingly, treatment of HeLa cells with two different Ca^2+^ ionophores, Ionomycin or A23187, increased MYPT1^S445^ phosphorylation and this increase was attenuated by NUAK1 inhibition (Figure 2e). Conversely, treatment with the calcium chelator BAPTA strongly reduced basal levels of phospho-MYPT1^S445^ (Figure 2f). Collectively, these data suggest that Ca^2+^ signalling activates NUAK1 in the absence of LKB1. Interestingly, treatment with the Ca^2+^ ionophore A23187 could partially rescue MYPT1^S445^ phosphorylation in the presence of NUAK1 inhibitor but not in the presence of the dual NUAK1/NUAK2 inhibitor, WZ4003 (Supplementary Figure S1A). A23187 also partially rescued MYPT1^S445^ phosphorylation upon suppression of either NUAK1 or NUAK2 but not both (Supplementary Figure S1B), together suggesting that NUAK2 is also activated by calcium signalling in the absence of LKB1.

### MYC drives increased PKC activity

**Figure 3:**
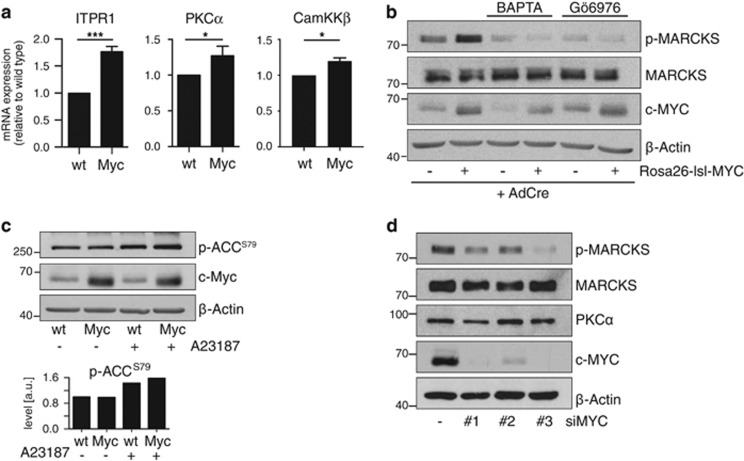
MYC selects for increased calcium signalling. (**a**) Total RNA isolated from WT or Rosa26-lsl-MYC MEFs harvested 24 h after infection with Adeno-CRE was analysed by Q-PCR for expression of the indicated transcripts. Mean and s.d. from biological triplicates shown. Statistical significance was determined by two-tailed unpaired *T*-tests. (**b**) Immunoblot from Adeno-CRE-infected WT or Rosa26-lsl-MYC MEFs as per (**a**) probed for expression and phosphorylation of the PKC substrate MARCKS. Where indicated, MEFs were treated with BAPTA (10 μm for 3 h) or Gö6076 (1 μm for 3 h) prior to lysis. (**c**) Lysates from WT and MYC-transformed MEFs, treated as indicated with 10 μm A23187 (10 min), probed with the indicated antibodies. Densitometry analysis shows normalized levels of p-ACC^S79^ from three immunoblots. (**d**) Immunoblot from HeLa cells upon depletion of MYC using three independent siRNAs versus non-targeting control (−), probed for p-MARCKS^S159/163^. All images are representative of at least three independent experiments, except (**d**) where *N*=2.

Calcium regulates multiple kinases including Ca^2+^/Calmodulin-dependent kinases 1–4; CamKK1 and 2; and conventional isoforms of protein kinase C (cPKC). Noting our previously described link between NUAK1 and MYC overexpression, we wondered if MYC might drive expression of a calcium-dependent kinase upstream of NUAK1. Oncogenic transformation of mouse embryo fibroblasts (MEFs) with MYC specifically increased expression of PKCα and CamKKβ, along with the inositol tri-phosphate receptor ITPR1, which regulates calcium release from the endoplasmic reticulum (Figure 3a). Notably, MYC was previously shown to bind the promoters of all three genes in diverse cell types, including MEFs.^30^ MYC overexpression strongly enhanced PKC activity, as measured by phosphorylation of the canonical PKC substrate MARCKS,^31^ and modestly but reproducibly enhanced Ca^2+^-dependent activation of AMPK (Figures 3b and c). MARCKS phosphorylation was suppressed by BAPTA or by treatment with the PKCα/β inhibitor Gö6976,^32^ suggesting that deregulated MYC specifically increases activity of Ca^2+^-dependent PKC isoforms (Figure 3b). HeLa cells express high levels of MYC^33^ and depletion of MYC in HeLa cells reduced p-MARCKS levels (Figure 3d), suggesting that this consequence of MYC overexpression is conserved across species.

### NUAK1 is activated by PKCα

**Figure 4:**
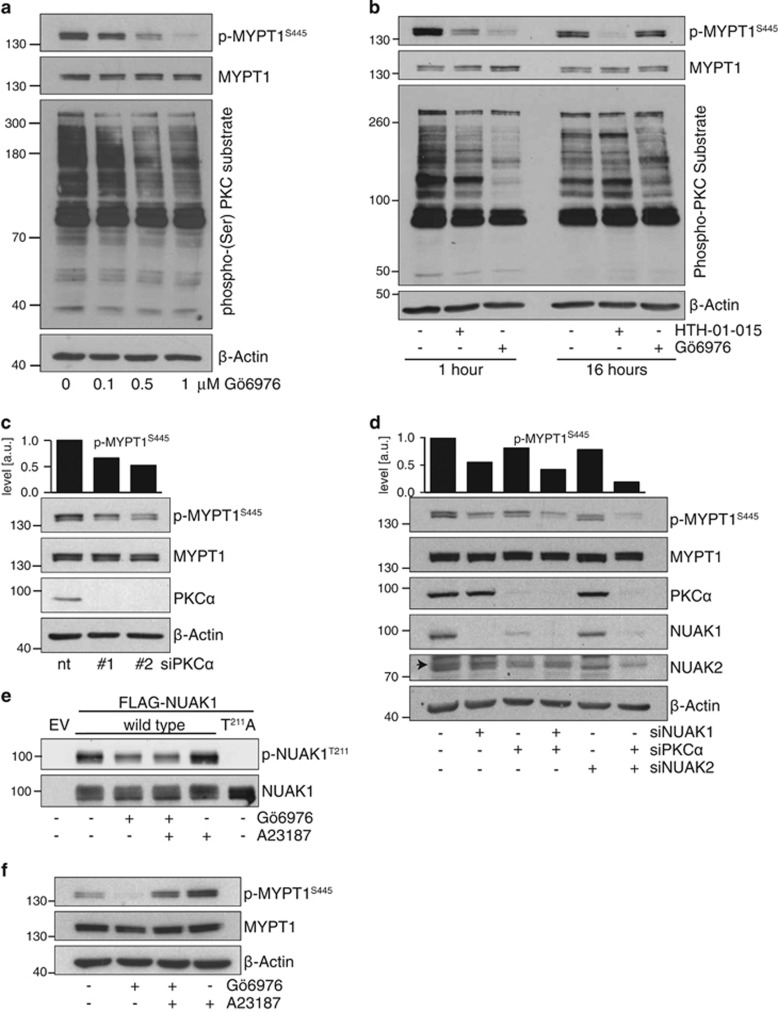
PKCα mediates calcium-dependent NUAK1 activation. (**a**) Lysates from HeLa cells treated with the indicated concentrations of Gö6976 for 1 h and probed with the indicated antibodies. (**b**) Lysates from HeLa cells treated with 1 μm Gö6976 or 10 μm HTH-01-015 for 1 or 16 h, probed with the indicated antibodies. (**c**) Lysates from HeLa cells transfected with PKCα siRNA and probed for phospho-S445 and total MYPT1 with densitometry of p-MYPT1 above. nt, non-targeting control siRNA. (**d**) Lysates form HeLa cells transfected with siRNA targeting PKCα and/or NUAK2 or NUAK1, probed with the indicated antibodies. Arrowhead indicates the correct band for NUAK2. (**e**) Anti-FLAG immunoprecipitates from HeLa cells transfected with FLAG-tagged WT or T211A mutant NUAK1, treated with 0.5 μm Gö6976 and/or 3 μm A23187, and probed with anti-phospho-NUAK1^T211^ antibody. (**f**) Lysates from HeLa cells pre-treated with 0.5 μm Gö6976 for 1 h prior to stimulation with 3 μm A23187, as per (**e**) and probed for phosphor-MYPT1^S445^. All images are representative of at least three independent experiments, except (**e**) where *N*=2.

We next asked if targeted suppression of PKC impairs NUAK1 activity. Inhibition of PKCα and β isoforms with Gö6976 strongly reduced p-MYPT1^S445^ in a dose-dependent manner (Figure 4a). Notably, this effect was transient, as p-MYPT1^S445^ levels rebounded within 16 h of Gö6976 treatment, and this was mirrored by a recovery in overall PKC activity (Figure 4b). SiRNA-mediated depletion of PKCα also reduced p-MYPT1^S445^ levels to a degree that was similar to NUAK1 inhibition but less than that observed after 1 h treatment with the highest concentration of Gö6976 tested, which may reflect promiscuity of the PKC inhibitor at this dose (Figure 4c; note that a lower concentration of Gö6976, 0.5 μm, was used for all subsequent experiments). No effect on p-MYPT1^S445^ was observed using siRNA targeting PKCβ1 (not shown).

In light of our data showing that both NUAK1 and NUAK2 contribute to Ca^2+^- induced MYPT1^S445^ phosphorylation, we asked if the effects of PKCα depletion were mediated by either NUAK1, NUAK2, or both. The reduction of p-MYPT1^S445^ achieved upon NUAK1 depletion was minimally influenced by co-depletion of PKCα (compare lane 2 with lane 4), consistent with a role for PKCα upstream of NUAK1. In contrast, suppression of MYPT1^S445^ phosphorylation by NUAK2 depletion was strongly enhanced by co-depletion of PKCα (compare lanes 5 and 6), suggesting that NUAK2 resides in a distinct pathway (Figure 4d). Interestingly, depletion of PKCα consistently reduced expression of NUAK1 (Figure 4d). This effect was observed using two independent siRNAs targeting PKCα and neither siRNA influenced NUAK1 mRNA levels (Supplementary Figure S2), strongly suggesting that the effect does not reflect off-target activity of the siRNAs used. Proteasome inhibition largely rescued NUAK1 levels upon depletion of PKCα, suggesting that PKCα promotes NUAK1 protein stability (Supplementary Figures S2B and C).

To examine the effects of acute calcium signalling on NUAK1 activation, we requisitioned an affinity-purified phosphopeptide antibody against T211-phosphorylated NUAK1, and overexpressed either wild type or T211A mutant, FLAG-tagged, NUAK1 in HeLa cells. In FLAG immunoprecipitates, the antibody strongly detected a band migrating at the correct size for NUAK1 only in lysates from WT but not from T211A mutant-overexpressing cells. Identical results were obtained using a commercial anti-phospho-AMPKα^T172^ antibody that cross-reacts with overexpressed phospho-NUAK1^T211^^34^ (Supplementary Figure S2). For both antibodies, the intensity of this band increased within 10 min of Ca^2^^+^ ionophore treatment and decreased upon acute treatment with PKC inhibitor (Figure 4e and Supplementary Figure S2). Examination of p-MYPT1^S445^ under the same conditions showed similar responses to Ca^2+^ ionophore and PKC inhibitor treatment with one important difference: whereas Ca^2+^ ionophore could partially rescue the effect of Gö6976 on MYPT1^S445^ phosphorylation (Figure 4f), no such rescue was evident in p-NUAK1^T211^ levels. Taken with the data above, these data suggest that Ca^2+^ signalling regulates NUAK1 in HeLa cells via activation of PKCα, while MYPT1^S445^ phosphorylation is regulated both via NUAK1 and via a distinct pathway involving Ca^2+^-dependent, Gö6976-refractory, activation of NUAK2.

### The PKCα–NUAK1 pathway supports viability of MYC-overexpressing cells

**Figure 5:**
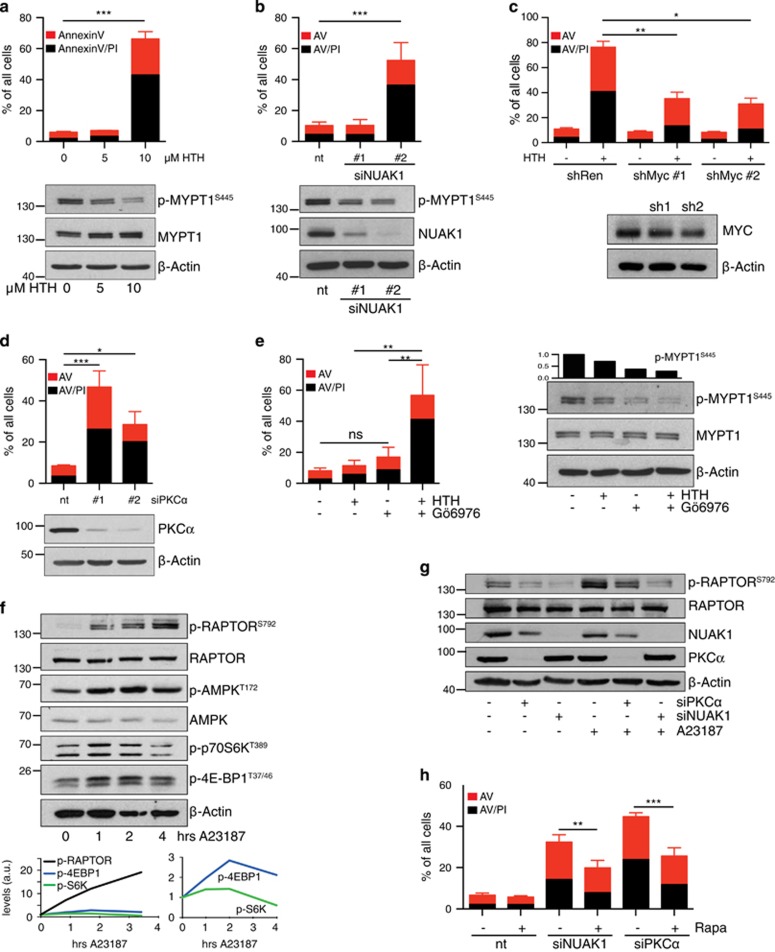
The PKCa–NUAK1 pathway supports viability of MYC-overexpressing cells. (**a**) Apoptosis induced in HeLa cells by the indicated doses of HTH-01-015, measured by FACS analysis of cells stained with Annexin V and propidium iodide (AV/PI) 48 h post-treatment: Red bars denote AV single-positive cells while black bars denote AV/PI double-positive cells (for **a**–**e**, **h**). Mean and s.d. of three independent experiments shown (**a**, **b**, **d**, **e**, **h**). Statistical significance was determined by one-way ANOVA, Tukey’s multiple comparison test (**a**–**e**). The immunoblot shows suppression of MYPT1 phosphorylation after 1 h treatment. (**b**) Apoptosis in HeLa cells induced by NUAK1 siRNA, measured 3 days post-transfection. Immunoblot shows NUAK1 and p-MYPT1 levels at 24 h. nt, non-targeting control siRNA. (**c**) Apoptosis induced by 10 μm HTH-01-015 in HeLa cells transfected with MYC shRNA where indicated. Mean and s.d. of technical triplicates from a representative (median) experiment shown due to wide inter-experimental variation in efficiency of MYC depletion. *N*=5. (**d**) Apoptosis induced in HeLa cells by PKCα siRNA, measured 3 days post-transfection. Immunoblot shows PKCα levels after 24 h. nt, non-targeting control siRNA. (**e**) Apoptosis induced in HeLa cells after treatment with 5 μm HTH-01-015±0.5 μm Gö6976 for 48 h. Immunoblot shows p-MYPT1^S445^ levels after 1 h drug treatment. (**f**) Dynamic response of the AMPK–mTORC1 pathway to calcium mobilization. HeLa cells were harvested at 0, 1, 2 and 4 h post-treatment with 3 μm A23187 and probed with the indicated antibodies. The graphs show densitometric measurements of p-RAPTOR^S792^, p-S6K^T389^ and p-4EBP1^T37/46^ from the blots shown, normalized to Actin. *N*=2. (**g**) Lysates from HeLa cells transfected with NUAK1, PKCα or non-targeting (−) siRNA for 24 h and stimulated with 3 μm A23187 for 10 min, as indicated, probed with the indicated antibodies. *N*=2. (**h**) Apoptosis induced by depletion of either NUAK1 or PKCα in cells treated for 48 h with 100 nm Rapamycin (Rapa) or DMSO vehicle control (−), measured by AV/PI FACS. Two-way ANOVA, Sidak’s multiple comparison test.

We previously showed that MYC-overexpressing cells require NUAK1 to sustain viability.^21^ HeLa cells express high levels of MYC and prolonged treatment (2 days) with 10 μm HTH-01-015 resulted in pronounced HeLa cell apoptosis (Figure 5a). Partial inhibition of NUAK1 with 5 μm HTH-01-015 was surprisingly well tolerated, suggesting that a threshold level of NUAK1 activity is sufficient to prevent cell death. Similar results were obtained using siRNA-mediated NUAK1 depletion, in that death was only induced upon very strong suppression of NUAK1 expression (Figure 5b). Death induced by 10 μm HTH-01-015 was significantly attenuated by reducing MYC levels with either of two MYC-targeting shRNAs (Figure 5c), consistent with our previous demonstration of MYC ‘dose-dependence’ for the synthetic lethal interaction with NUAK1.^21^ Consistent with a role for PKCα upstream of NUAK1, depletion of PKCα with either of two siRNAs also drove pronounced HeLa cell apoptosis (Figure 5d), while treatment of HeLa cells with Gö6976 significantly enhanced killing by a sublethal dose of NUAK1 inhibitor (Figure 5e). Note that Gö6976 treatment alone did not kill HeLa cells, likely owing to the transient nature of PKC inhibition by this compound (Figure 4b).

We asked if death induced upon loss of PKCα mechanistically mirrored that induced by loss of NUAK1. Under conditions of energetic stress, cancer cells activate a metabolic checkpoint in order to limit mTORC1-driven macromolecular synthesis, via phosphorylation of RAPTOR-Ser^792^ by AMPK.^35^ Failure to engage this checkpoint results in death of stressed cells^24, 36^ and our previous work showed that NUAK1 is required for efficient checkpoint activation.^21^ Dynamic analysis of this checkpoint in HeLa cells revealed a complex response to Ca^2+^ ionophore, with p-RAPTOR^S792^ increasing steadily over time whereas phospho-S6K^T389^ and phospho-4EBP1^T37/46^ levels, downstream of mTORC1, rose initially before declining (Figure 5f), consistent with Ca^2+^ simultaneously activating the mTORC1 pathway as well as the inhibitory AMPK–Raptor pathway.^37^ Importantly, depletion of either PKCα or NUAK1 reduced both basal and Ca^2+^-activated phosphorylation of RAPTOR-Ser^792^, suggesting that failure to efficiently engage the metabolic checkpoint may contribute to death in both instances (Figure 5g). Consistent with this hypothesis, treatment of HeLa cells with the mTORC1 inhibitor Rapamycin significantly rescued cells from death induced by depletion of either NUAK1 or PKCα, and the degree of rescue was similar in both instances (Figure 5h). Although these data do suggest that other downstream pathways likely contribute to cell death, they strongly support the core observation that NUAK1 and PKCα act in a similar manner to support cell viability.

### NUAK1 regulates RAPTOR via both AMPK-dependent and independent mechanisms

**Figure 6:**
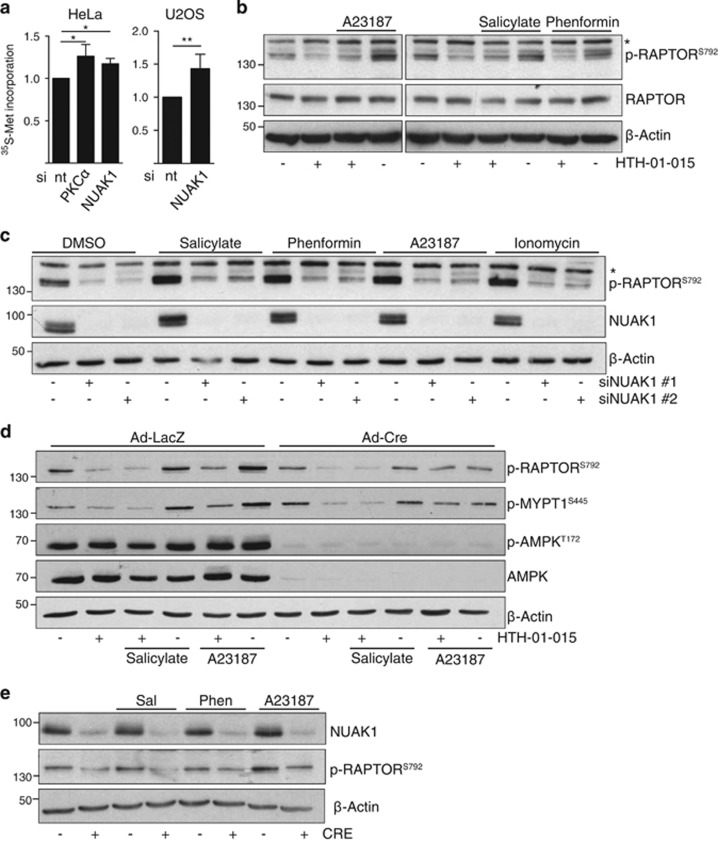
NUAK1 regulates RAPTOR via AMPK-dependent and independent mechanisms. (**a**) Measurement of protein synthesis (methionine incorporation) in HeLa (left panel) and U2OS (right panel) cells transfected with non-targeting (−), NUAK1 and PKCα siRNA. Mean and s.d. from three independent experiments shown. Statistical significance was determined by one-tailed unpaired *T*-test. (**b**) Lysates from U2OS cells pre-treated with 10 μm HTH-01-015 for 1 h, where indicated, prior to treatment with 6 μm A23187 (10 min), 10 mm salicylate (1 h), 10 mm phenformin (1 h) or DMSO vehicle and blotted for phospho-S792 and total RAPTOR. *N*=3. The asterisk denotes a nonspecific band in the p-RAPTOR panel (**b**, **c**). (**c**) Lysates from U2OS cells transfected where indicated with siRNA targeting NUAK1 and treated with 10 mm salicylate (1 h), 10 mm phenformin (1 h), 6 μm A23187 (10 min), 3 μm Ionomycin (10 min) or DMSO vehicle, blotted for p-RAPTOR^S792^. *N*=3. (**d**) Lysates from immortalized *Prkaa1*^*FL/FL*^*;Prkaa2*^*FL/FL*^ double floxed MEFs, infected overnight with Adeno-LacZ or Adeno-CRE and treated as per (**c**) with AMPK activators in the presence or absence of 10 μm HTH-01-015, blotted with the indicated antibodies. *N*=2. (**e**) Lysates from primary *Nuak1*^*FL/FL*^ MEFs stably expressing Cre-ER were treated overnight with 100 nm 4-OH-Tamoxifen (+) or vehicle control (−) prior to stimulation as per (**d**, **e**) with AMPK activators, then immunoblotted for p-Raptor^S792^. *N*=2.

Confirming the requirement for NUAK1 to restrain mTORC1 activity, ^S35^-Methionine labelling showed increased protein translation in NUAK1-depleted HeLa and U2OS cells (Figure 6a), as shown previously.^21^ We therefore examined RAPTOR regulation by NUAK1 in greater detail. Activation of AMPK by Ca^2+^ ionophore (A23187), phenformin or salicylate in U2OS cells all lead to increased RAPTOR^S792^ phosphorylation. In contrast with the selective requirement for NUAK1 during AMPK regulation of ACC, RAPTOR^S792^ phosphorylation was reduced by NUAK1 inhibition under all conditions examined (Figure 6b). Depletion of NUAK1 also significantly reduced both basal and AMPK-activated RAPTOR^S792^ phosphorylation, confirming the specificity of this effect (Figure 6c). Inhibition of NUAK1 reduced AMPK-dependent RAPTOR^S792^ phosphorylation in immortalized *Prkaa1*^*FL/FL*^*;Prkaa2*^*FL/FL*^ double floxed MEFs. Strikingly, phospho-RAPTOR^S792^ was still detectable in the same MEFs after CRE recombinase-mediated deletion of AMPKα1 and α2, albeit at reduced levels, and NUAK1 inhibition further reduced detection, indicating that NUAK1 can regulate RAPTOR in the absence of functional AMPK (Figure 6d). Accordingly, deletion of NUAK1 in Nuak1^FL/FL^ MEFs also reduced both basal and AMPK-activated RAPTOR^S792^ phosphorylation (Figure 6e). Together these data show that efficient restraint of mTORC1 via inhibitory phosphorylation of RAPTOR requires both NUAK1 and AMPK.

## Discussion

**Figure 7:**
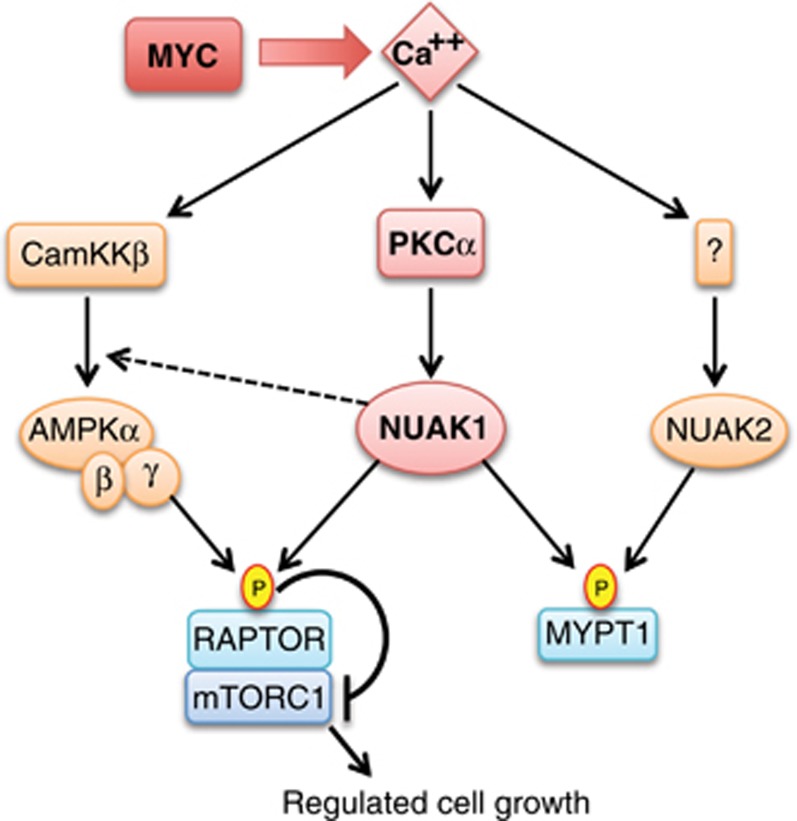
Diagram of calcium regulation of NUAK1, NUAK2 and AMPK.

Suppression of NUAK1 is synthetic lethal with MYC overexpression, suggesting that NUAK1 may present an attractive target for treatment of MYC-driven cancers.^21, 38^ A thorough understanding of the signal transduction context of NUAK1 will be crucial to determine if such a strategy is feasible in human subjects. Here we show that NUAK1 is active in HeLa cells despite the absence of LKB1. We show modulation of NUAK1 activity by calcium perturbation, and present evidence that PKCα participates in NUAK1 activation in response to Ca^2+^ signalling. Importantly, Ca^2+^-dependent activation of the AMPK–mTORC1 metabolic checkpoint requires both PKCα and NUAK1, and depletion of either drives pronounced apoptosis, suggesting a positive role for this pathway in tumour maintenance. Our specific findings are summarized in Figure 7.

It is widely thought that the tumour suppressive function of LKB1 is mediated by one or more of the AMPK-family kinases.^27, 39^ Loss of LKB1 would thus be predicted to result in loss of ARK activity, downstream. Accordingly, deletion of *Stk11*, encoding Lkb1, in wild-type MEFs was shown to suppress activity of AMPK and all related ARKs, as measured in cell-free kinase assays using a peptide substrate optimized for AMPK.^1^ However, several of the ARKs, including Nuak1, showed only weak activity towards the peptide used, suggesting it was a suboptimal substrate for these kinases. Indeed, subtle differences in peptide substrate sequences have revealed distinct preferential phosphorylation patterns of AMPK and MARK kinases.^40^ Thus, *in vitro* kinase assays with a one-size-fits-all peptide substrate likely fail to accurately reflect physiological ARK activity in cells. Additionally, several independent groups have definitively shown that AMPK is directly phosphorylated by CamKK2, reflecting an alternative pathway to AMPK activation.^25, 26, 41, 42^ Activation of AMPK by CamKK2 is particularly important in prostate cancer and in the physiological regulation of skeletal muscle and vascular endothelial cell function.^43, 44, 45^ Interestingly, the ARK SIK2 was recently shown to be activated by an as-yet unidentified Ca^2+^-dependent kinase in Ovarian cancer cells.^46^ Our demonstration that NUAK1 and NUAK2 are similarly regulated by Ca^2+^-dependent signalling thus fits an emerging pattern of calcium regulating multiple ARKs, either alongside or in the absence of LKB1. This regulation may have particular relevance in LKB1-deficient disease settings.

Our data speak to the complexity of signal transduction through AMPK, NUAK1 and the related ARKs. Indeed, AMPK is often discussed as if it were a single entity. Rather, up to 12 different permutations of trimeric AMPK complexes can assemble from the 2α, 2β and 3γ-encoded subunits, not accounting for splice variants.^22^ It is likely that the different AMPK complexes may respond differentially to distinct upstream stimuli, and indeed in terms of their activity towards specific downstream substrates. Our demonstration of a specific requirement for NUAK1 in Ca^2+^-dependent AMPK activity towards ACC, and a more general requirement for NUAK1 in AMPK activity towards RAPTOR, point towards a highly contextual requirement for NUAK1 and may indicate that NUAK1 modulates the activity of a specific subset of AMPK complexes. On top of this, the 11 related ARKs can exhibit both overlapping and private substrate specificities. This is reflected by our demonstration of an AMPK-independent role for NUAK1 in RAPTOR regulation, and by phosphorylation of MYPT1 by NUAK1, NUAK2 and potentially by additional ARKs. Consistent with this, we also find Ca^2+^-dependent phosphorylation of the canonical AMPK substrate ACC even after complete suppression of CamKKβ-dependent AMPK activity in HeLa cells. Clearly, considerably more work will be needed to disentangle these complex signalling networks.

Whereas calcium has long been recognized to drive MYC expression^47^ and more recently to regulate MYC function,^48, 49^ the reciprocal regulation of calcium signalling by MYC has not garnered much attention. MYC was shown to increase calcium signalling during B-cell differentiation by suppressing expression of the calcium exporter PMCA.^50^ ChIP-SEQ analysis has revealed MYC binding to the promoters of *ITPR1-3*, *PRKCA* and *CamKK2* in diverse cell types,^30^ consistent with our observation that MYC promotes expression of these genes. The pronounced increase in phosphorylation of the PKC substrate MARCKS compared with the much more modest effect of MYC overexpression on PKCα levels suggests that regulation of this pathway by MYC is only partially explained by the observed transcriptional effects. Nevertheless, our data do suggest that MYC actively selects for increased cellular sensitivity to calcium, and does so in part to promote NUAK1 activity, maintain metabolic homoeostasis and thereby sustain cell viability. The relative contribution of calcium signalling to NUAK1 activation likely depends on several factors including the strength of calcium signalling, whether LKB1 is present or absent and, if present, the relative levels of PKCα and LKB1 upstream.

MYC is a paradigm driver of apoptosis when expressed at high levels^51, 52^ whereas conventional PKC isoforms inhibit apoptosis in many cell types.^53^ Suppression of PKCα or β induces apoptosis,^54, 55, 56^ whereas overexpression has been shown to suppress death induced by MYC or withdrawal of IL3.^57, 58^ Both PKCα and β isoforms have been shown to promote Ser^473^ phosphorylation of AKT,^59, 60^ which inhibits canonical MYC-induced apoptosis, primarily via suppression of pro-apoptotic BH3 protein expression/function.^61, 62, 63^ Importantly, we showed previously that MYC-overexpressing cells continue to require NUAK1 even when active AKT is overexpressed,^21^ pointing to a role for NUAK1 in protecting tumour cells from non-apoptotic cell death and suggesting that calcium and PKCα may govern multiple pathways that promote tumour cell survival. Targeted suppression of these pathways may thus have therapeutic benefit in multiple cancers where MYC is deregulated.^64^

## Materials and methods

### Cell culture

The identity of all cell lines was verified using an in-house cell line validation service. HeLa and U2OS cells were maintained in Dulbecco’s modified Eagles’s medium containing 4.5 g/l glucose, 1% glutamine, 100 U/ml of streptomycin, 100 U/ml of penicillin, 10% fetal bovine serum and incubated at 37 °C in 5% CO_2_. Primary MEFs were isolated from mouse embryos (wild type; Rosa26-lsl-Myc; Nuak1^FL/FL^) at E13.5 days and cultured as above except for incubation in 3% oxygen. All cell lines were routinely tested for mycoplasma contamination and were validated by STR profiling using an approved in-house validation service (CRUK-BICR). Wild type, Rosa26-lsl-MYC MEFs were infected with 300 multiplicity of infection of Adeno-Cre replication-incompetent virus (University of Iowa) to induce MYC expression. Nuak1^FL/FL^ MEFs were infected with retrovirus expressing tamoxifen-inducible Cre-ER and selected on puromycin. SV40 T antigen-immortalized *Prkaa1*^*FL/FL*^*; Prkaa2*^*FL/FL*^ double floxed MEFs were generously provided by Russell Jones, McGill University. For transient transfection, HeLa cells were plated on 10 cm diameter dishes and transfected with 3 μg of DNA (FLAG-NUAK1wt, FLAG-NUAK1T211A or empty vector) using Lipofectamine 3000 (Thermo Fisher, Waltham, MA, USA) and lysed 48 h post-transfection. For protein translation measurements, cells were cultured for with 30 μCi/ml ^35^S-Methionine label (EasyTag from Perkin Elmer, Beaconsfield, UK) for 30 min and total protein was precipitated using a final concentration of 12.5% trichloroacetic acid. Scintillation (Ecoscint, Thermo Fisher) was counted for 2 min.

### Chemicals and antibodies

Phenformin, Sto-609, Rapamycin, phosphatase inhibitor cocktails (P0044 and P5726), protease inhibitor cocktail (P8340) and MG132 were purchased from Sigma-Aldrich (Irvine, UK); HTH-01-015 from Cambridge Bioscience (Cambridge, UK); A23187, Ionomycin and A769662 from Abcam (Cambridge, UK); WZ4003, Gö6976 and BAPTA-AM were purchased from Tocris (Bristol, UK). Antibodies recognizing ACC phospho-Ser79(#3661), total ACC (#3676), Raptor phospho-Ser792(#2083), total Raptor (#3661), AMPK phospho-T172(#2535), total AMPK (#2532), total MYPT1 (#8574), PKCα (#2056), NUAK1 (#4458), phospho-(Ser) PKC substrate (#2261), MARCKS phospho-Ser159/163 (#11992) were purchased from Cell Signalling Technologies (Danvers, MA, USA); anti-FLAG (#F1804), anti-β-Actin (#A5441) were from Sigma-Aldrich; anti-MYPT1 phospho-Ser445(#S508C) and anti-NUAK2 (#S225B) were from the MRC PPU, Dundee, UK; anti-MARCKS (#ab72459) anti-Histone H2B (#ab1790), anti-Vinculin (#ab129002) and anti-c-Myc (#ab32072) were purchased from Abcam. The phospho-T211 NUAK1 antibody was generated by Eurogentec (Liege, Belgium) against the phosphopeptide KFLQT^PO3^FCGSPLY. The antibody was affinity purified from reactive serum using the same phosphor-peptide after counter-selection with non-phosphorylated peptide. In addition to the results shown, the antibody was further validated by loss of signal upon siRNA-mediated depletion of NUAK1. Secondary antibodies coupled to horseradish peroxidase anti-mouse and anti-rabbit were purchased from GE Healthcare (#NA931 and #NA934; Chicago, IL, USA), and anti-sheep was from Pierce (#31480; Thermo Fisher).

### RNA interference

HeLa cells were passaged 12 h before transfection and transfected at 70% confluency using Lipofectamine RNAiMAX (Thermo Fisher) with the following siRNA from Qiagen (Manchester, UK): non-targeting control (1022076), NUAK1#1 (SI00108388), NUAK1#2 (SI00108388), PKCα#1 (SI00605934), PKCα#2 (SI00605927), NUAK2 (SI02660224), MYC#1 (SI00300902), MYC#2 (SI02662611), MYC#3 (SI03101847). shRNA against human MYC and a non-targeting control (Renilla) were designed by and purchased from Mirimus Inc. (Woodbury, NY, USA): ShMYC1702—CGCCTCCCTCCACTCGGAAGGA; shMYC1891—CTGAGTCTTGAGACTGAAAGAT. HeLa cells were transfected with 3 μg of shRNA-encoding plasmid using Lipofectamine 3000. After transfection, cells were treated and analysed as for figure legends.

### Quantitative real-time PCR

RNA was isolated by Trizol and was reversed transcribed using QuantiTect Reverse Transcription Kit (Qiagen), according to the manufacturer’s instructions. Real-time quantification was performed using SYBR Green Fast Mix (VWR, Lutterworth, UK) with C1000 thermal cycler (Bio-Rad, Watford, UK). Primers for NUAK1 (forward, 5’-ccgctcactgatgtaatcgt; reverse, 5’-gtcatctctcaaccatcctcat), ACTIN (forward, 5’-ccaaccgcgagaagatga; reverse, 5’-ccagaggcgtacagggatag) ITPR1 (forward, 5′-GAAGGCATCTTTGGAGGAAGT-3′ reverse, 5′-ACCCTGAGGA-AGGTTCTG-3′), PKCα (forward, 5′-CAAGGGATGAAATGTGACACC-3′ reverse, 5′-CCTCTTCT-CTGTGTGATCCATTC-3′), CaMKKβ (forward, 5′-GGAGGTCGAGAACTCAGTCAA; reverse, 5′-CATGGTCTTCACCAGGATCA) and β2m (forward, 5′-ACCTCCATGATGCTGCTTAC-3′ reverse, 5′-GGACTGGTCTTTCTATCTCTTGTAC-3′) were obtained from IDT (Leuven, Belgium).

### Immunoprecipitation and immunoblotting

FLAG-NUAK1 wild type, mutant (T211A) or empty vector transiently overexpressed HeLa cells were rinsed with ice-cold phosphate-buffered saline and then lysed in Lysis Buffer containing 50 mm Tris-HCl (pH 7.5), 1% NP-40, 0.27 m sucrose and phosphatase/protease inhibitors. Cell lysates (1 mg) were incubated overnight at 4 °C with anti-FLAG M2 Affinity gel (Sigma, Irvine, UK; A2220). Immunoprecipitated were washed twice with Lysis Buffer containing 0.15 m NaCl, twice with 50 mm Tris-HCl (pH 7.5) plus phosphatase inhibitors and resuspended in sodium dodecyl sulphate sample buffer. For whole-cell extracts, cells were rinsed with ice-cold phosphate-buffered saline and then lysed *in situ* with lysis buffer containing 150 mm NaCl, 50 mm Tris (pH 7.5), 1% NP-40, 0.5% sodium deoxycholic acid, 1% sodium dodecyl sulphate plus protease and phosphatase inhibitor cocktails. Lysates were then sonicated to reduce viscosity and diluted in sodium dodecyl sulphate sample buffer. Immunoprecipitated and whole-cell extracts were resolved by sodium dodecyl sulphate–polyacrylamide gel electrophoresis, transferred to nitrocellulose membranes for subsequent incubation with primary antibodies overnight at 4 °C. Densitometry analysis of individual immunoblots was performed using ImageJ (NIH, Bethesda, MD, USA).

### Cell death analysis

HeLa cells were treated or transfected as for figure legends and on the day of the analysis the supernatant was collected, cells were rinsed in phosphate-buffered saline and harvested by trypsinization. Cells were then centrifuged at 300 *g* for 5 min at 4 °C and pellet incubated in 200 μl Annexin V binding buffer (10 mm HEPES pH 7.4, 140 mm NaCl, 2.5 mm CaCl_2_) containing APC-Annexin V (Biolegend, San Diego, CA, USA) for 10 min at room temperature. Propidium iodide was added prior to analysis by FACSCalibur (BD Biosciences, Wokingham, UK) flow cytometry.

### Statistical analysis

Raw data were uploaded into Prism (Graphpad, La Jolla, CA, USA) or Excel (Microsoft, Reading, UK) spreadsheets for generation of graphs. All experiments were performed on at least three occasions, except where noted, and mean and s.d. values from biological replicates are presented. Statistical significance was determined by *T*-test and one-way or two-way ANOVA as per figure legends. * denotes *P*<0.05; **<0.01; ***<0.001.
