# Supplementary material for: Calcium signalling links MYC to NUAK1
Source: Oncogene. 2017 Nov 6;37(8):982–92. doi: 10.1038/onc.2017.394 (PMC5815498; doi:10.1038/onc.2017.394)
Supplement: Supplementary Information [file onc2017394x1.pdf]

Supplemental Information

Figure S1: Calcium activation of NUAK1 and NUAK2 in HeLa cells

A) Lysates from HeLa cells treated with 6μM A23187 on the presence or absence of the NUAK1-selective inhibitor HTH-01-015 (10μM for 1 hr) or the NUAK1/NUAK2 inhibitor WZ4003 (10μM for 1hr), probed for phosphor-S445 and total MYPT1. **B)** Lysates from HeLa cells infected with siRNA targeting NUAK2 or non-targeting (-), and treated as above with 6μM A23187 and/or HTH-01-015 (10μM for 1 hr), blotted for phosphor-S445 and total MYPT1. Densitometry of the p-S445 MYPT1 blot shown above. Note that the first 4 lanes are the same images as shown in Main Figure 2C. N=3 experiments.

Figure S2: Regulation of NUAK1 protein levels by PKCα

A) Immunoblot of NUAK1 protein expression in HeLa cells upon depletion of PKCα, followed by treatment with proteasome inhibitor MG132 (20μM for 5hrs). **B)** Mean and SD fold decrease in NUAK1 protein expression after PKCα depletion in the presence or absence of proteasome inhibitor MG132. N=3. NS = not statistically significant. **C)** Q-PCR measurement of NUAK1 mRNA levels in HeLa cells transfected with either of 2 siRNAs targeting PKCα or non-targeting control. Error bars show SD of technical triplicates. N=2. **D)** Anti-FLAG immunoprecipitates from HeLa cells transfected with FLAG-tagged WT or T211A mutant NUAK1, treated with 0.5μM Gö6976 and/or 3μM A23187, and probed with anti-phospho-AMPK^{T172} antibody, which cross-reacts with overexpressed NUAK1. N=2.

Figure S1

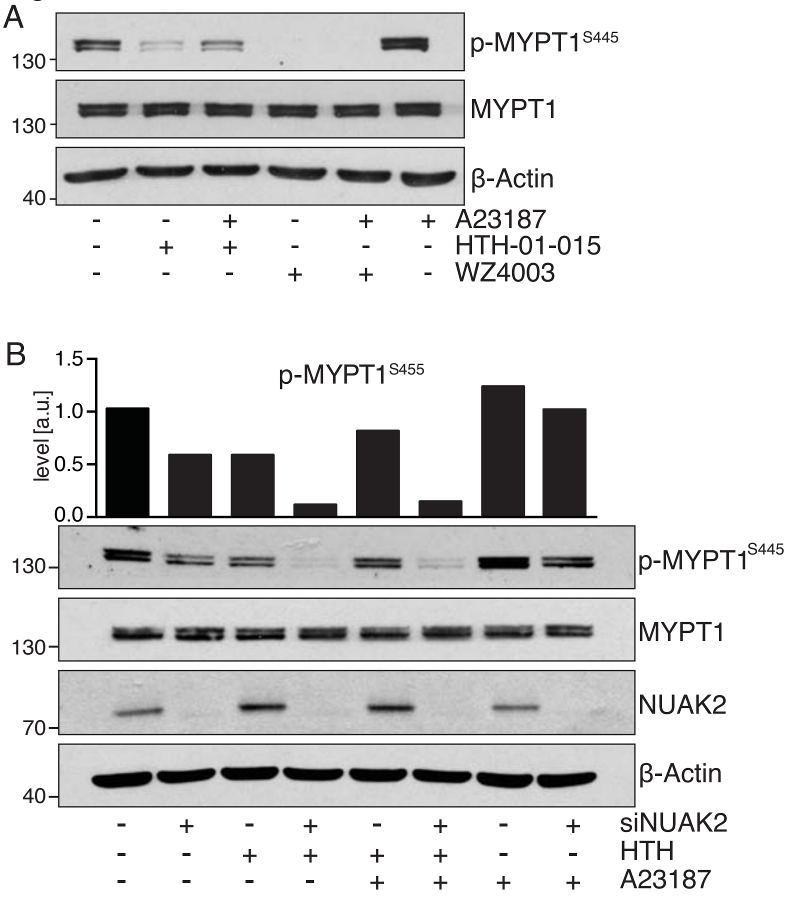
A
p-MYPT1S445
MYPT1
β-Actin
130
130
40
A23187
HTH-01-015
WZ4003
B
p-MYPT1S455
level [a.u.]
1.5
1.0
0.5
0.0
p-MYPT1S445
MYPT1
NUAK2
β-Actin
130
130
70
40
siNUAK2
HTH
A23187

Figure S2

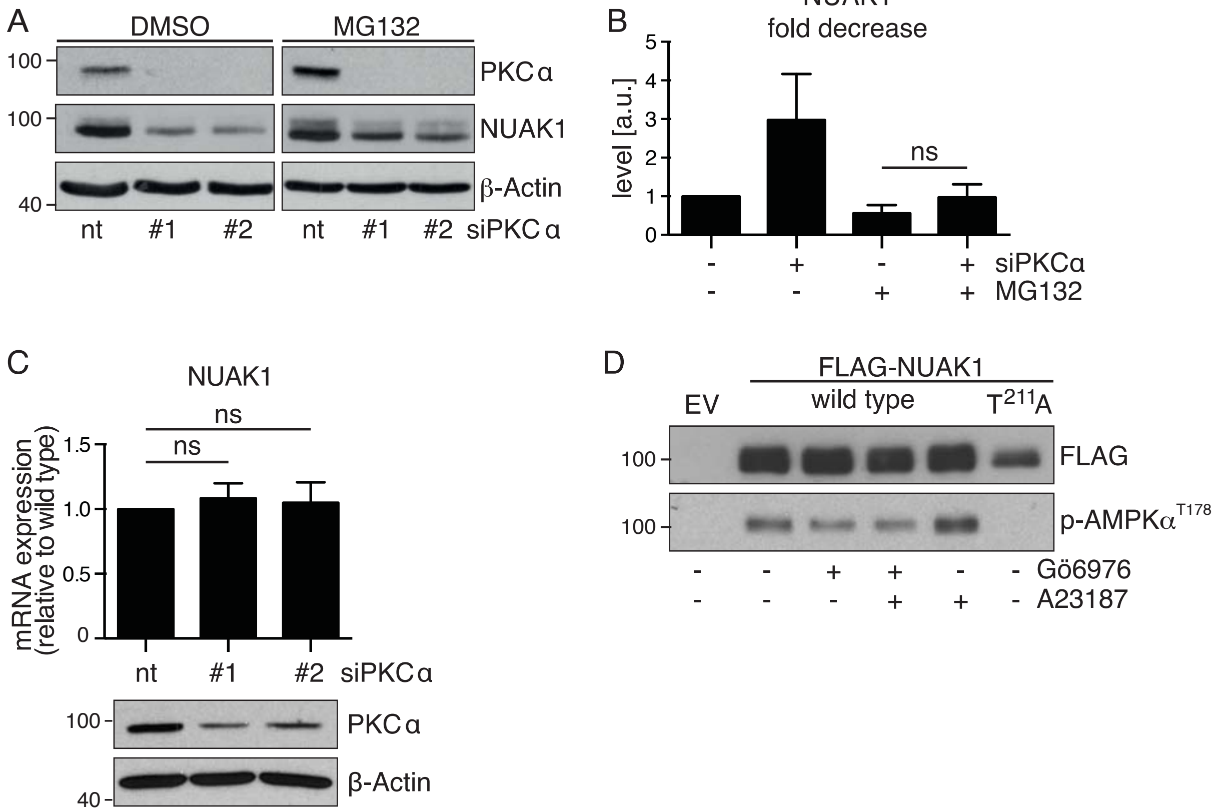

A
DMSO
MG132
100
PKCα
100
NUAK1
β-Actin
40
nt #1 #2 nt #1 #2 siPKCα
B
NUAK1
fold decrease
level [a.u.]
5
4
3
2
1
0
ns
- + - + siPKCα
- - + + MG132
C
NUAK1
ns
ns
mRNA expression
(relative to wild type)
1.5
1.0
0.5
0
nt #1 #2 siPKCα
100
PKCα
β-Actin
40
D
FLAG-NUAK1
EV
wild type
T211A
100
FLAG
100
p-AMPKα T178
- - + + - - Gö6976
- - - + + - A23187
